# The Use of Assistive Technology to Promote Care of the Self and Social Inclusion in Patients with Sequels of Leprosy

**DOI:** 10.1371/journal.pntd.0004644

**Published:** 2016-04-28

**Authors:** Fátima Beatriz Maia, Enéas Rangel Teixeira, Gislaine Valeria Silva, Maria Katia Gomes

**Affiliations:** 1 Departamento de Terapia Ocupacional, Universidade Federal do Rio de Janeiro, Rio de Janeiro, Rio de Janeiro, Brazil; 2 Escola de Enfermagem Aurora de Afonso Costa, Universidade Federal Fluminense, Niteroi, Rio de Janeiro, Brazil; 3 Programa de Pós-Graduação em Clínica Médica, Hospital Universitário Clementino Fraga Filho e Departamento de Medicina de Família e Comunidade/Faculdade de Medicina da Universidade Federal do Rio de Janeiro, Rio de Janeiro, Rio de Janeiro, Brazil; Fondation Raoul Follereau, FRANCE

## Abstract

**Background:**

This study is about the contribution of occupational therapy inside a rehabilitation group, and we focus on the autonomy of patients with disabilities due to leprosy. There are few studies on the use of assistive technology by leprosy patients; to our knowledge, none of them aim to have a subjective approach of care. Our purpose was to analyze the repercussions of assistive technology on autonomy of care of the self in patients with sequels of leprosy.

**Methods:**

A qualitative, descriptive exploratory study with a semi-structured interview and a field observation as a research method was conducted between November 2014 and February 2015 at a University Hospital in Rio de Janeiro.

**Findings:**

Eight patients from the service of Occupational Therapy were interviewed, and 44 hours of observation were performed. Interviews followed a semi-structured script and a field journal was used to take notes. Analysis was conducted by the hermeneutic approach. Costs were obtained after a global cost analysis of the fixed and variable expenses and direct and indirect costs to the manufactured products with an amount of 100 dollars. Results were grouped according to the following categories: contribution of the adapted devices for the care of the self and feelings and sensations provoked by the use of self-help devices. The reports revealed feelings, perceptions and meaningful contents about the social, familiar and individual dimensions, also the stigma coupled with leprosy. However, forms of re-signification were elaborated.

**Conclusions:**

Assistive technology empowers the subject to perform care of the self and promotes social inclusion.

## Author Summary

This study is about occupational therapy and we focus on the autonomy of patients with disabilities due to leprosy. Our purpose was to analyze the repercussions of assistive technology on the autonomy of care of the self in patients with sequels of leprosy We performed a qualitative, descriptive, exploratory study between November 2014 and February 2015 at a university hospital in Rio de Janeiro, and interviewed eight patients. Analysis was conducted by the hermeneutic approach. The following categories were studied: contribution of the adapted devices for the care of the self and feelings and sensations provoked by the use of self-help devices. The reports revealed feelings, perceptions and meaningful contents about the social, familiar and individual dimensions, also the stigma coupled with leprosy. Assistive technology is an approach with a powering potential that provides tools for the subject's care of the self and promotes social inclusion.

## Introduction

The interest on the topic emerged after the implantation of the service of Occupational Therapy in a University Hospital in Rio de Janeiro, among the interdisciplinary team for Prevention, Physical and Surgical Rehabilitation in Leprosy.

Once we started investigating the functional limitations of patients in their activities of daily living (ADL), we noticed that these limitations could be reduced with assistive technology, by suggesting the use of adaptations that ease the occupational performance.

With the purpose of achieving a better knowledge of the possibilities of helping the process of making in the human being as its impact on the subject, we first investigated the routine of the leprosy patients, to assess the tools they do or do not use during their ADLs which would require adaptations. This initial investigation in the practice of the Occupational Therapy motivated the elaboration of this research.

Occupational Therapy presents resources, means or technologies to rescue the inclusion and an independent life. Assistive Technology is composed of resources and services that help providing or improving functional skills. It can be considered an indispensable tool to provide the inclusion and the integration of people with deficiency, with an amplification of the context of the society [1].

This technology is designed especially for people with disabilities with the aim of providing a framework for considering how products and environments can be designed to accommodate the broadest range of users [2].

Borg and Larson (2009) have observed that more attention has been given to the preventing role of assistive devices compared to the facilitating role. The focus of assistive devices facilitating functioning has been on mobility aspects of care of the self and domestic life, which might also facilitate activities and participation in other life areas such as work and employment. To achieve a better utilisation of assistive devices there should be a training of related professionals and staff on user-involved design [3].

The intersection between technology and subjectivity rescues the appreciation of the participation of the subject in the process of treatment and rehabilitation. The use of the assistive technology in the life of the subject influences the care of the self, that is the ability to manage their own life.

This paper contributes to the theoretical and practical concepts of the health care practices, research and innovation in this area, also the changes in the teaching of the occupational therapy in both inter and multidisciplinary approaches.

## Methods

### Design

This study employed a descriptive exploratory design, with a qualitative aspect, in which we used a semi-structured interview and field observation as research tools.

### Data gathering

The room for the conduction of the research was the ambulatory of Occupational Therapy created in November, 2012, in a University Hospital placed at Ilha do Governador in Rio de Janeiro. The hospital provides assistance, teaching and research in several areas. The leprosy program has begun in 1990 and it has become a reference for diagnosis, treatment, surgeries and rehabilitation of leprosy.

The interviews were conducted, taped in a digital recorder and fully transcripted by the authors.

The interview script searched for the following aspects: report of the repercussion of the assistive technology in the autonomy of the subject's care of the self and instrumental aspects; value of the technology and subjective dimensions.

Based on the Hermeneutic approach, we've established some wide questions to be answered by the patients, as follows below:

1. How many adapted utensils have you received and how many of them could you incorporate into your routine?
2. How was the care of the self before and how is it now, after receiving the adaptations?
3. How important was the adaptation for your life?
4. What was the impact on the practical aspects of daily life?
5. How did you notice yourself?
6. How did you manage your own life?
7. Which feelings do you feel when talking about limitations and during the use of adaptations?

The devices to be produced were determined after a motor and sensitive evaluation of the patient. During it, it was possible to detect their physical condition and to prevent risks associated with the absence of sensation. During the study, patients were asked about the difficulties in their regular activities and the importance given to such disability.

All patients received at least one adapted utensil and were trained for the use of the following instruments: forks, knifes, spoons, mugs, wooden spoon, toothbrushes, shavers, pen and scissors. The work tools adapted were: screwdriver, monkey wrench and grip pliers. Orthoses for ulnar claw were also built.

A total of 44 hours of observation were performed and registered in a field journal, that also contained the evaluation of the participants, production of the equipment and training of the adaptations.

During the observational period, information about the professional care, relation aspects and process of selection of the patients for the interviews were gathered. During this period, we also took notes on the individuals' nonverbal language.

The main objective of adaptations was to allow performing actions, especially the ones that the subject values, letting them to become more independent and fulfilled. The training process was also a moment to discuss the adhesion of the use of devices, and all patients were able to perform tasks in a new, adapted manner.

**Fig 1:**
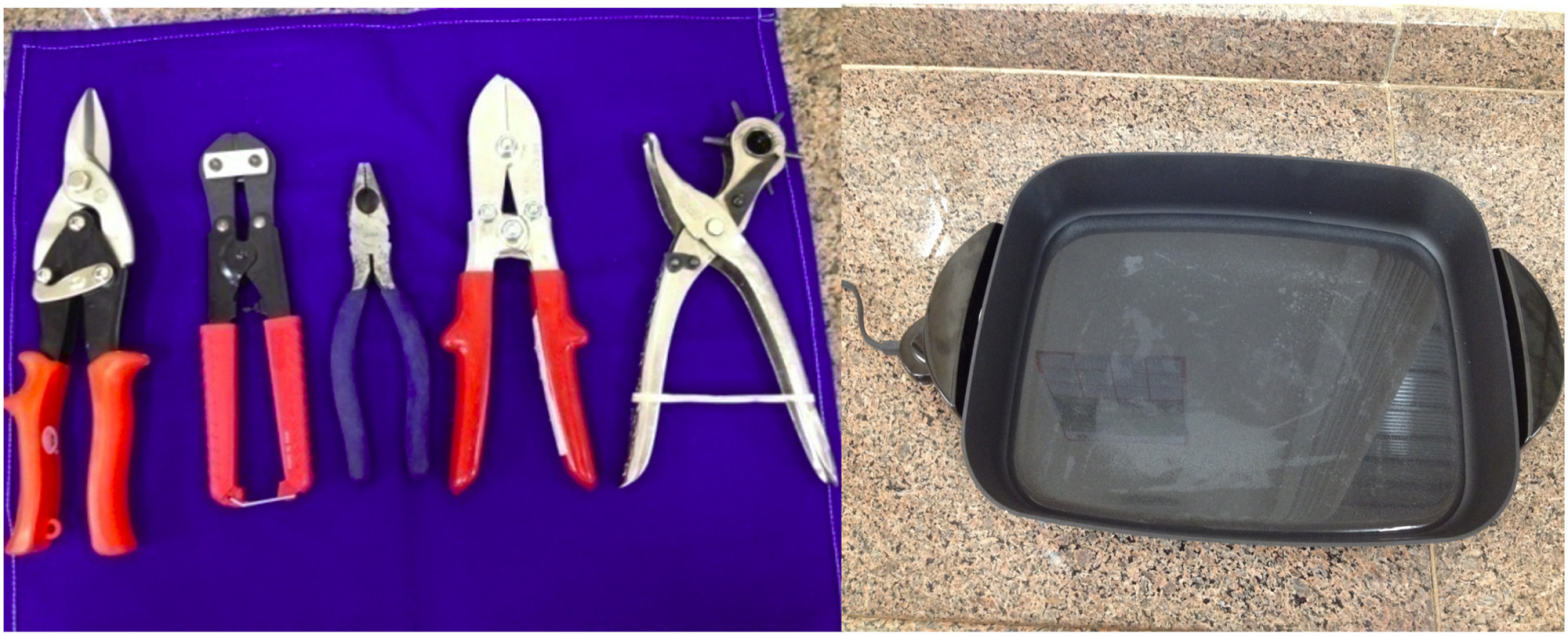
Materials used to develop adaptations (left) and heat up termomoldable (right).

Patients brought their own utensils from home to be adapted, such as flatwares and tools. This way, we respected patient's culture, did not change their lifestyle and also made them responsible for their own treatment. Materials for adaptations such as lining, foam, PVC, thermomoldable and others achieved a total cost of USD100, and are shown in Fig 1.

### Ethics statement

This research was submitted and approved by the Human Research Ethics Committee from the Hospital Universitário da Universidade Federal Fluminense, with and acceptance letter from the coparticipant institution HUCFF/UFRJ, in the date of August 8th, 2014, by under the number 774.178, register CAAE: 30503914.5.0000.5243 according to the Resolution 466/12 of the National Health Council that controls researches with human beings. All participants have provided a signed informed consent.

## Results

### Participants

After observation, 8 patients were selected. They were older than 15 years of age, with grade 2 disability[4]. They all accepted the use of the assistive technology. There were 13 patients in the service, but 5 were excluded due to sequels of other diseases and cognitive and/or mental disturbs (Table 1).

**Table 1:** Characteristics of the sample.

Patient	Age (years)	Gender	Marital Status	Monthly Income (minimum wage)	Disease duration (years)	Sequels	Adapted Utensils	Training period (sessions)
**1**	58	M	Married	3	28	Bilateral ulnar and median claw	Fork, Knife, Spoon, Shaver	6
**2**	58	M	Single	1	19	Bilateral ulnar and median claw	Fork, Knife, Spoon, Shaver, Toothbrush	8
**3**	27	M	Single	0	5	Bilateral ulnar and median claw	Fork, Knife, Spoon, pen, Toothbrush, positioning orthesis	8
**4**	61	M	Married	1	11	Bilateral ulnar and median claw	Fork, Knife, Shaver, Toothbrush, screwdriver, monkey-wrench, grip pliers	7
**5**	59	M	Married	1	4	Right Ulnar claw, loss of visual acuity	Mug, toothbrush, knife, screwdriver	5
**6**	53	M	Single	2	3	Bilateral Ulnar claw	Fork, knife, shaver, toothbrush, positioning orthesis	9
**7**	65	F	Married	2	12	Bilateral ulnar and median claw	Mug, scissors, toothbrush, wooden spoon, knives.	7
8	50	M	Widower	1	9	Bilateral ulnar, median and radial claw	Mug, toothbrush, spoon, knife, fork.	5

Legend: M = male; F = female; Minimum wage = the lowest remuneration that employers may legally pay to workers, equivalent to R$880 per month (USD220).

The content of the interviews and the observations evidenced verbal and non-verbal expressions, emotional and meaningful contents regarding their social, family and individual dimensions and stigma related to leprosy. Unequal job opportunities, difficulties related to the physical limitation and the way patients became dependent on their family members were present in the reports and generated body expressions and gestures to be reviewed in the Discussion topic.

The content of the research was divided into two categories: contribution of adapted devices for the care of the self; and feelings and sensations provoked by the use of the adapted instruments (Table 2).

**Table 2:** Reports of the patients according to the categories.

Categories	Expressive words from reports	Data collected from observation (reports and non-verbal expressions)
1. Contribution of self-help devices to the care of the self	✓ It is really helpful	✓ I won't ask for help to open a bottle anymore
	✓ Easier to grasp	✓ I can cut with this hand (as performs the movement)
	✓ Trying to learn	✓ See? I shaved by myself.
	✓ Now I can hold a bottle	✓ It is so much easier to eat outside
	✓ I can do things with more precision	✓ I still do it differently, but now I do.
	✓ It has helped me in daily living situations	✓ Positive hand gestures of achievement were present during training sessions
	✓ Now I can also practice at home	
	✓ It is easier to eat, but harder things are still difficult to cut.	
2. Feelings and sensations provoked by the use of adapted instruments	✓ I feel relieved	✓ I seem almost normal doing things. I can do what other people do (big smile present)
	✓ I feel like I won.	✓ It is a relief not to ask for help of other people(silence and emotion. Patient reported a difficult family relation). Another similar report: Do you know what it is like to ask help for people who don't want to help you?
	✓ The more I can do without depending on others, the better it is	✓ Everything we do is more difficult (as shows deformed hands, frowns and expresses sadness)
	✓ I don’t' eat so I don't disturb the others	✓ Now I can press screws alone, as I did before the disease(expression of joy as performs the action in a new manner)
	✓ We participate	✓ Social gathering: now I can have my meals anywhere. Another patient feels embarrassed: I don't use it outside, it draws too much attention.
	✓ I feel like the others	✓ Satisfaction to perform care of the self: It is wonderful to brush my teeth with a toothbrush(winning gesture with open arms)
	✓ I become normal	✓ Other expressions were noted, such as: embarrassment for not performing simple movements, impatient during training sessions.
	✓ My self-esteem is higher	✓ Most common gestures were: thumbs up, a '”v” for victory with fingers and many laughs.
	✓ It brings happiness and joy	
	✓ I was sad because I couldn't do anything, now I can.	
	✓ This is not good to wear, but a lot of people need it.	
	✓ Sometimes I call names, because I am so angry	
	✓ It reduces my difficulties, which is good.	

## Discussion

Discussion aims to connect the technology and the subjectivity in the process of care in an interdisciplinary approach using the Hermeneutic analysis to interpret the data, according to the categories of the study.

### Contribution of the self-help devices to the care of the self

The limitations acquired during ADL that were a topic for this research, such as grabbing, cutting, writing, screwing and performing other care of the self activities have led us to do more than build and train the devices. Such care practice approximated our group from the patients as we experienced the limitations and the search for ways to surpass them. Once we enter someone's routine, we must create an atmosphere of reception, approach and intimacy.

Several gestures emerged together with the statements. “this finger (index) disrupts me, it is very small and I can't hold things”(patient 4). One must consider that anesthetic, amputated or with re-absorption hands are limited in their functionality.

**Fig 2:**
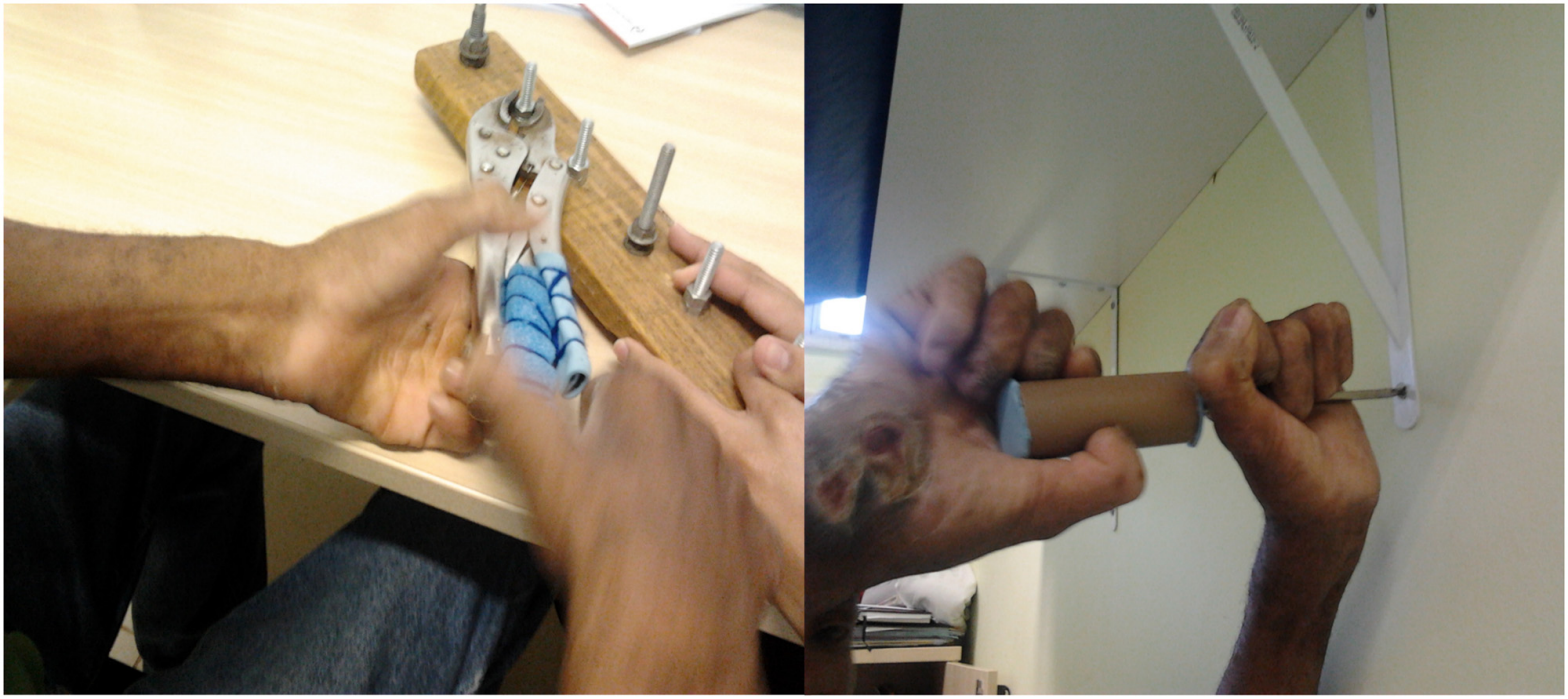
Training of labor activity with adapted tools.

The patient above demonstrated how he performed actions, despite of his injured hands, by a reelaboration of his possibilities through adaptation as develops his own way of solving problems, such as “when the screw head is small, I place it in a magnet, which makes it easier to get…” (Fig 2).

The contributions of adaptations for the patients' lives also aimed to prevent accidents, such as “…the knife sometimes cuts my hand.. I've never cut myself….the adaptation is good….the hilt is thicker…” (patient 1).

The use of adaptations promotes a distribution of the prehension forces, enhances functionality and reduces the influence of the deficiency into an independent performance of functional activities.

**Fig 3:**
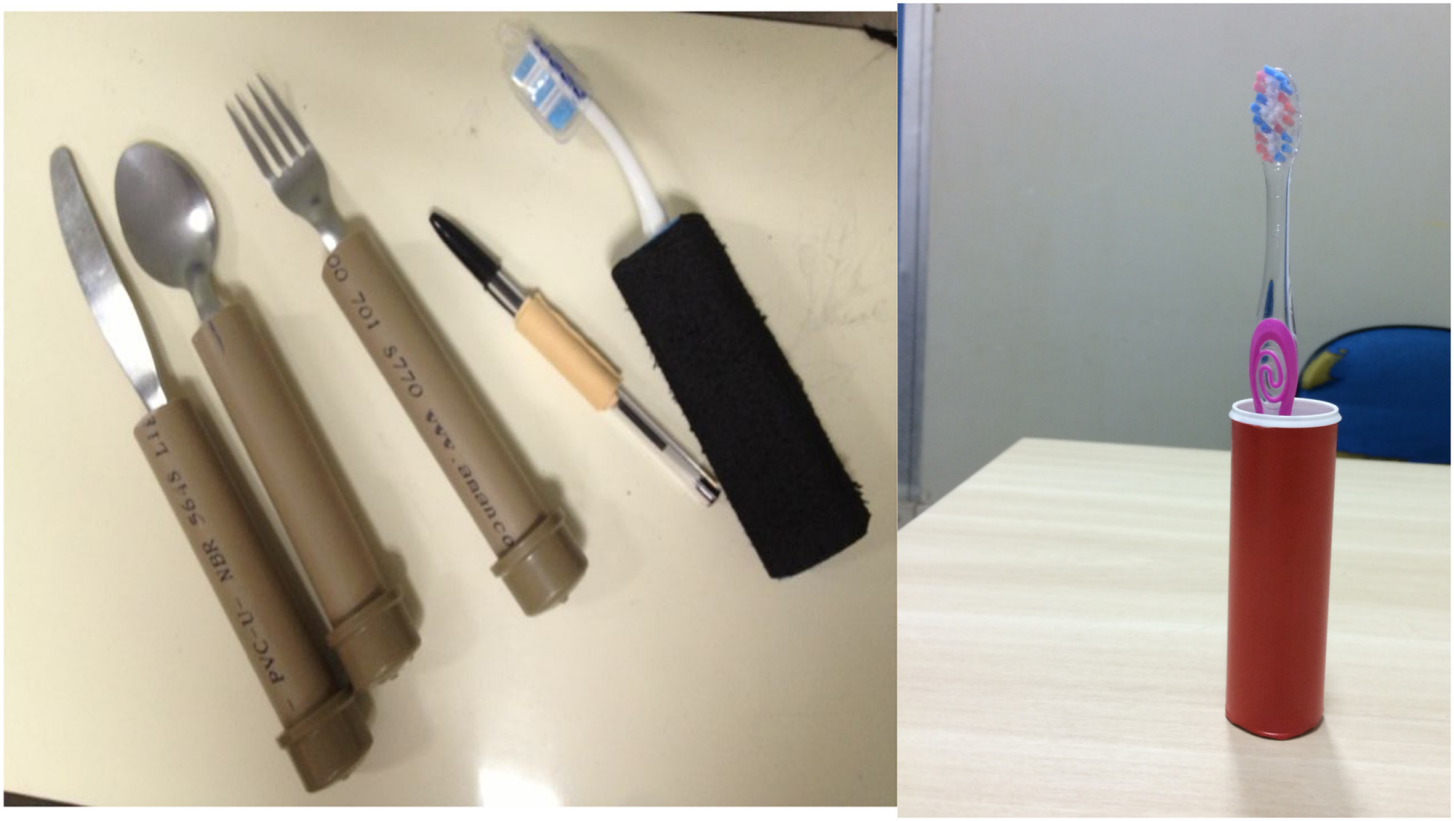
Flatware (left) and toothbrush (right) adapted with PVC.

To be safe while performing an action was interpreted by the patients as a possibility for autonomy and care of the self. Patient 7, who received a wider wooden spoon refers a safer sensation by being away from the steam of the pots. Even in the absence of pain, no patient wants to have an injury, which can be seen as sign of self-neglect. To facilitate self-care, patients received flatware and toothbrushes (Fig 3).

Our results along with the search for the autonomy in the care of the self, establishes a connection with Foucault's care of the self idea. Foucault [5] affirms that “To take care of the self is to know oneself, one's soul, as soul-subject, the element that identifies to the divine.”

*“The entire surface of the care of the self is occupied by this requirement of self-knowledge which*, *as you know*, *takes the form of the soul's grasp of its own being*, *which it carries out by looking at itself in the mirror of the intelligible in which*, *precisely*, *it has to recognize itself”* [5]

The adaptation of utensils provided a possibility of safety and independence during the recovery process, which are autonomy actions for the subject's care, frequently expressed as the examples “I' will be able to write again”(patient 3), “…now I can hold the bottle and easily drink water”(patient 6). Such reports connect themselves with the amplification of the subjective and bodily possibilities, as the adaptations can be considered an extension of the body and a facilitator of the restoration of the human making process.

When the patients reported their perception of the use of the adapted utensil in their ADL, it was observed a re-elaboration of the making, with a new sense of the care of the self, as registered in the following sentences: “to be able to finish studying, to have a high school degree is a relief, a victory.”(patient 3), “The more we can do without any help, the better” (patient 7).

Therefore, to facilitate one's care of the self actions involves aspects such as: their way of living, choices, feelings, representations and other elements that act in the building of the way of being. It occurs an ethical implication of respecting the autonomy of the subject, rescue their dignity and providing social interactions.

When observing the frequent expressions of joy and satisfaction facing the rescue of the ADL, it is observed an aesthetics of existence, which, according to Foucault[6] aims for the constitution of one's self as the artesian of the beauty of one's own life.

This aesthetic dimension shows that the taste for the new form of living, the satisfaction for the autonomy, brings up the promotion of health and quality of life in a context of treatment and rehabilitation. A patient's report was “Do you know how it feels to ask someone who doesn't want to help?”(patient 3) followed by “This adaptation increased my self-steem” (patient 5). “It brings happiness and joy”(patient 1), and another one affirmed “We feel safer inside” (patient 7).

### Feelings and sensations provoked by the use of adapted instruments

Foucault [6] affirms that the hermeneutics analysis is a joint point between the body and the history. It evidences the body as marked by the history, and denotes the need to interpret the interpretation of the routine activities incorporated. In the field of the hermeneutics analysis there are the corporeality and its historical representations, throughout the subject's sensibility.

**Fig 4:**
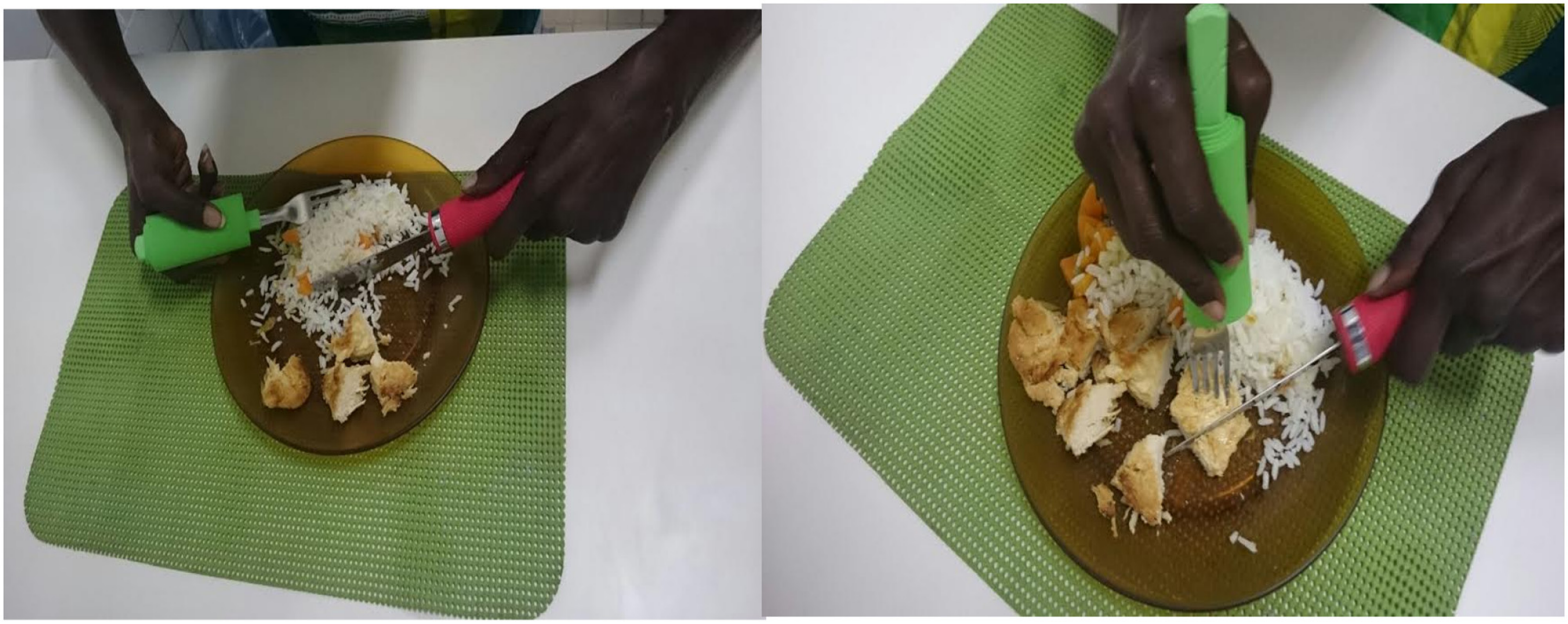
Training of ADL with adapted fork and knife.

The unique reports show the need for acceptance and integration in the society, which they have been denied due to the body sequels and social stigma historically produced. “We just feel equal, right?…other people, the normal ones, then I become normal” (patient 1). “How come someone else can cut and I can't?”(patient 5), “I used to watch people sitting at a table holding knives and forks, and I felt powerless, you know?.. Now I don't, when I cut, I also participate”, “I was sad because I couldn't do anything” (patient 1) (Fig 4).

Subject is submitted to mercy of distinct factors linked to someone else by control and dependence; and tied to his own identity by a conscience or self-knowledge. Despite one's individual perception of unique and owner of its own destiny, we also have a tendency to totality and fullfillness [7].

Social indifference leads to a feeling of sadness, described as a psychic suffering, which must be carefully identified and discussed when working with such clients. That is due to an affective sequel between the bodily representation and the subject's history. Once we faced such situation, we could understand it and help the subject when developing its own the care of the self. The assistive technology was applied to help achieving autonomy, social participation and a will to live, reducing one's suffering. Once we discussed the differences, emotion emerged along with a search for comprehension, as the following quotes affirm: “Doctor, you don't know what it feels like!!” (patient 2) and indignation and sadness looks manifested. This reflexion leads us to believe that technical knowledge is not enough to comprehend the complexity, one must also listen, welcome and follow the process of care of the subject.

Some overcome sentences manifested as “It's like we are reborn, learning it all again”(patient 3), “I couldn't get anything with this hand, but now I can”(patient 4), “…it's a good exercise to learn what I couldn't do before”(patient 7). That evidences the desire, the possibility to make choices and avoid placing oneself as a victim as reinventing through the limits and possibilities of life.

**Fig 5:**
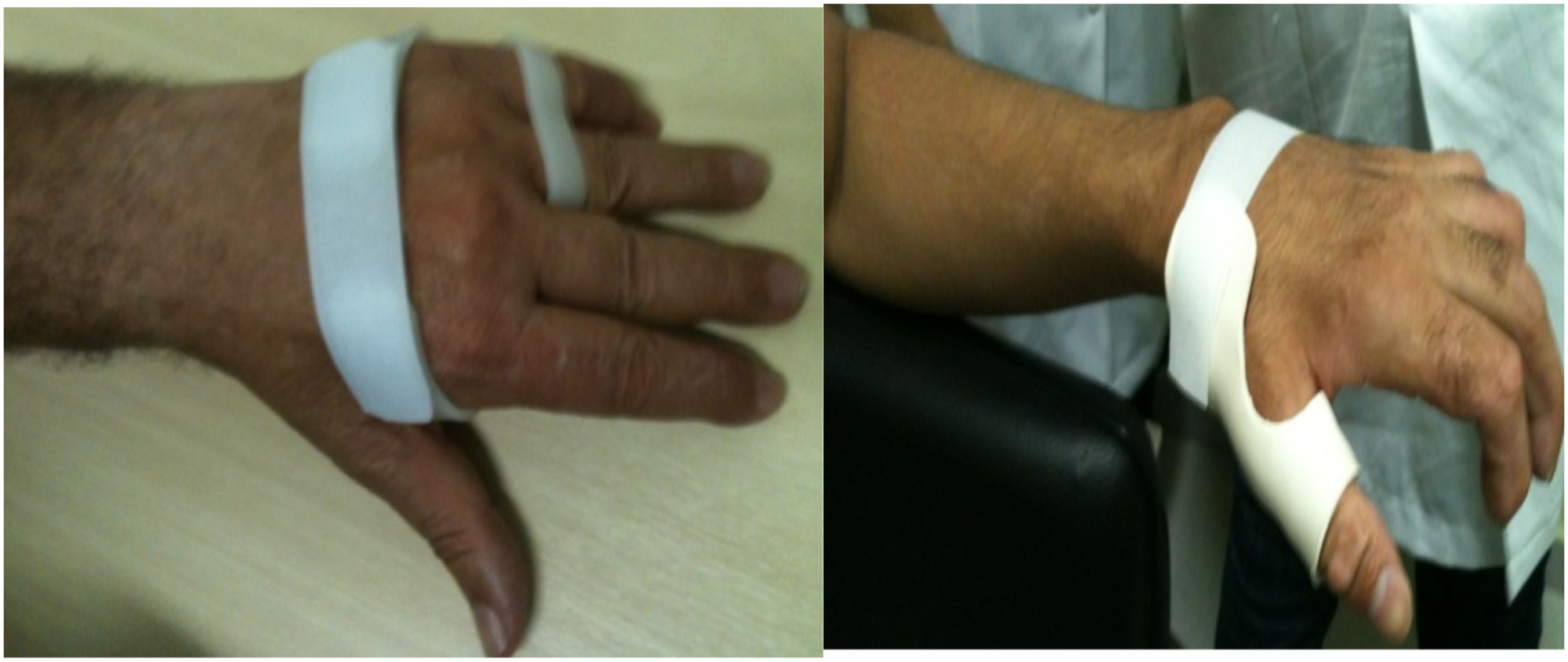
Orthoses for wrist extension during grasping (left) and positioning of the thumb (right).

These are evidences for relearning and reconstruction of the making (Fig 5). It is fundamental, during this period, to deal the patient's resistance, to avoid them from keeping in the process and to maximally enjoy the opportunities for a nurturing and interactive relearning. Such changing moment was observed through manifestations of joy, satisfaction and surprise throughout the possibilities. Also gestures and lines of conquer emerged, accompanied by hopeful smiles and looks, provided in a comprehensive and conscious manner by the assistive technology.

Although the sensation of happiness was manifested, feelings of anger and indignation were also observed. Objective and subjective limitations appeared in a few reports: “This is not good to use, but a lot of people need it.”(patient 8) “I become so angry that I call names, sometimes” “…I don't eat to avoid disturbing others”(patient 2). Sometimes movements could not be performed even in the use of the adapted devices, so frustration appeared. This topic was also discussed in our sessions, without giving up the new possibilities. To live with pleasure and the lack of it, love and hate are indispensable parts of the human learning process, and can't be removed from the health care.

The use of self help devices causes social awkwardness and can attract as much attention as the deformities caused by leprosy. This is probably one of the reasons of the low adhesion of the use of adapted devices when in public. Some patients affirm the use of adapted equipment at a restaurant as an award. Others demonstrate shame and report to be more observed in the use of the adaptations. “I don't use it outside, people look at it”(patient 2). The truth is that society does not easily tolerate differences. One will always search for the appropriate behavior, the desire for uniformness. This problematic was also discussed, the adhesion of the use of the orthoses and adaptations, with a focus on the adapted action, and how this subjectively affects the person.

The hermeneutics analysis points the need for the interpretation to constantly interpret itself, especially the re-signification and re-codification of the messages and signs. The sign begins to hold the contradictions, oppositions, tensions, negativity and positivity required to play a dialectic of interpretation [8].

Hermeneutic procedure allows to “speak again” and provides a wide possibility to new destiny on the speech.

When a patient with injured hands sadly says “Everything we do is more difficult”(patient 4) and follows that by “I won't need to ask for help to open a bottle anymore”(patient 7) it is important to allow them to listen how they can give a new meaning to this impotence and tiredness towards their difficulties.

The professional must recognize this endless building process. As Paulo Freire says [9], “The unfinished or inconclusive is part of the vital experience of the human being. Where there's life, there is something unfinished”, it can be realized that this impulses self knowledge and the appearance of latent desires, such as fantasies, dreams and other subconscious manifestations.

This is a perennial, never conclusive movement. We don't walk only forward, but we can walk back, go deeper, walk in any direction. The same occurs for the interpretations, they need to relate through a complex net of symbols. It has been a priority in our research to guarantee this environment of welcome and rebuilding.

The main findings of our research appeared in the rediscovered feelings during the use of the self-help devices, through expressions of overcome or frustration during the training sessions, smiles, moments of silence, and strengthening of the therapeutic bonding between patient and health care professional.

### Conclusions

Assistive technology is a worthy therapeutic instrument in the care of people with special needs. It must be involved in a context of a symbolic net that involve the body, he function, social role and the society when dealing with the inclusion, among other existential and relational aspects.

The lack of more information on the use of the assistive technology in leprosy has led us to continue this research. The number of cases is high, and new forms of sensitive and effective care must be searched, to change the profile of the rehabilitation offered, providing forms to rescue the autonomy and independence.

To affirm that the patient is autonomous, free and conscious of their choices, facing the embarrassing economic and social moments that difficult the access to information, it is fundamental to equal the relation of power between patient and health care professional. Aspects as one's desires, potentials and rights must be valued.

When analyzing the contribution of the devices in the routines and care of the self, feelings and sensations provoked, it was verified how important it is to provide instruments to the patients that allow the care of the self and how much this interferes in the preservation of the autonomy and social inclusion of the individual. The use of the devices does not represent a high cost for one's treatment, but has an impact in the rescue of the abilities lost with the evolution of the leprosy disabilities, which should be funded as research.

We must consider through gestures and speaking that the body of the patient with leprosy expresses scars of a collective history of suffering. Each subject express its own issues towards body and symptoms, but the conditions at the moment can not be ignored. The perception that the assistive technology represents a tool with a changing power for the rescue of these patients' identity leads to believe in new possibilities of care.
